# Ivabradine and Milrinone—Bridge to Recovery in New HFrEF With Low-Output Heart Failure

**DOI:** 10.1155/2024/5278240

**Published:** 2024-09-02

**Authors:** Lekha Racharla, Lucas Gitzel, Max Joseph, Desire Guthier, Nael Hawwa

**Affiliations:** ^1^ Lehigh Valley Heart and Vascular Institute Lehigh Valley Health Network, Allentown, Pennsylvania, USA; ^2^ Department of Internal Medicine Lehigh Valley Health Network, Allentown, Pennsylvania, USA; ^3^ Morsani College of Medicine University of South Florida Health, Tampa, Florida, USA; ^4^ Department of Cardiovascular Medicine University of Florida, Gainesville, Florida, USA

**Keywords:** heart failure recovery, heart failure remission, inotrope, ivabradine, reverse remodeling

## Abstract

In patients with newly diagnosed heart failure with reduced ejection fraction (HFrEF) who are on a downward clinical trajectory, options are mostly limited to left ventricular assist device and transplantation. However, in those with factors favorable for reverse remodeling, every effort should be made to promote native myocardial recovery. We present three patients with newly diagnosed severe HFrEF, NYHA Class 3–4 symptoms, and low cardiac output with and without organ malperfusion. Ivabradine and/or home milrinone were used during their tenuous hemodynamic period as a bridge to optimize guideline-directed medical therapy (GDMT), device therapy, and eventual reverse remodeling. Evidence of structural heart improvement continued far beyond 1 year.

**Summary**

• Understand maladaptive sinus tachycardia.
• Understand the utility of inotrope as bridge to optimizing GDMT.
• Understand the potential of late reverse remodeling beyond 1 year

## 1. Introduction

In the setting of new-onset heart failure (HF) with reduced ejection fraction (HFrEF), the goal is always recovery or remission. A dilemma presents itself in the following scenarios: (1) clinical and hemodynamic status precludes the necessary time to allow for recovery, (2) intolerance of beta-blockers, and (3) persistent maladaptive sinus tachycardia.

Although in these situations, evaluation for a left ventricular assist device (LVAD) or heart transplantation is a viable option, given the medical, psychosocial, and financial implications of such modalities, the following less conventional alternatives exist: (1) home inotrope as a bridge to allow time for guideline-directed medical therapy (GDMT) titration and device therapy and (2) ivabradine to address maladaptive sinus tachycardia until hemodynamic status improves to allow beta-blocker titration.

**Figure 1:**
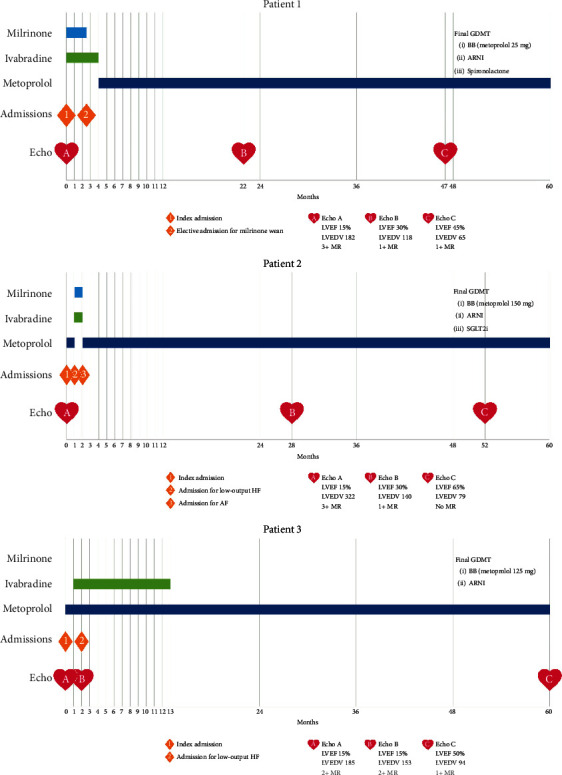
Patient chronologic events. LVEF = left ventricular ejection fraction; LVEDV = left ventricular end-diastolic volume; MR = mitral regurgitation; GDMT = guideline-directed medical therapy.

We present three patients faced with these clinical dilemmas who had successful outcomes with the abovementioned strategies (Figure 1).

## 2. Patient 1

A 59-year-old female was admitted with weeks of HF symptoms progressing to resting symptoms. She was found to have pulmonary edema but no organ malperfusion. Workup revealed left bundle branch block (LBBB) >150 ms, severely dilated left ventricle (LV), ejection fraction (EF) 15%, normal right ventricle (RV) size, mildly reduced RV function, and normal coronaries. The etiology was possibly idiopathic. Initial hemodynamics were right atrial pressure 17 mmHg, pulmonary artery pressure 61/31/41 mmHg, pulmonary capillary wedge pressure 31 mmHg, and Fick cardiac index 1.2 L/min/m^2^. She required milrinone, but despite optimization with decongestion and vasodilators, she failed multiple attempts to wean over the following 8 days, including clinical decompensation. She was deemed inotrope-dependent. Despite days of improved hemodynamics, she had a persistent resting sinus tachycardia of 100–110 bpm. Ivabradine 5 mg twice daily was added and titrated to 7.5 mg twice daily 2 days later, with improvement in heart rate (HR) to 70–90 bpm. CRT-D was performed in an expedited fashion for the following reasons: (1) waiting at least 3 months on GDMT was not an option as she was on a trajectory toward needing advanced HF therapies, (2) LBBB >150 ms is a strong predictor of CRT benefit, and (3) protection from malignant ventricular arrhythmia with home inotrope.

The patient was discharged on milrinone 0.25 mcg/kg/min and GDMT including sacubitril–valsartan and spironolactone. GDMT was titrated. She had clinical improvement, and 2.5 months later, she had an elective admission for hemodynamic-guided inotrope wean, which was successful. Ivabradine was transitioned to metoprolol a few months later. There was slow evidence of reverse remodeling. LVEF remained severely reduced on the echocardiogram 22 months after the initial diagnosis, with significant improvement at 47 months. The patient remained in NYHA Class 1, not requiring loop diuretics, and did not have a HF admission in the following 5 years.

## 3. Patient 2

A 47-year-old female with noninsulin-dependent diabetes was admitted with weeks of HF symptoms. Workup revealed LBBB >150 ms, severely dilated LV, EF 18%, no edema or late gadolinium enhancement (LGE) on MRI, mildly dilated RV, mildly reduced RV function, and normal coronaries. The etiology was possibly idiopathic. The patient was diuresed and started on GDMT.

She was readmitted 1 month later with low output HF, acute kidney injury (AKI), and congestive hepatopathy. Creatinine was 1.51 mg/dL (*ref* < 1.10), and AST was 669 U/L (*ref* < 41). There was no precipitating event. Initial hemodynamics were right atrial pressure 16 mmHg, pulmonary artery pressure 45/26/32 mmHg, pulmonary capillary wedge pressure 26 mmHg, and Fick cardiac index 1.6 L/min/m^2^. She required milrinone and aggressive diuresis, with improvement of symptoms and resolution of AKI and congestive hepatopathy. Despite medical optimization with decongestion and vasodilators, she failed multiple attempts to wean milrinone over the following 6 days. Despite days of improved hemodynamics, she had a persistent resting sinus tachycardia of 110–120 bpm. Ivabradine 5 mg twice daily was started with an improvement in HR to 80–90 bpm. CRT-D was performed in an expedited fashion for similar reasons to Patient 1.

The patient was discharged on milrinone 0.375 mcg/kg/min and GDMT including sacubitril–valsartan, spironolactone, and empagliflozin. She had clinical improvement but was readmitted 1 month later with atrial fibrillation (AF) with rapid ventricular response. Given improved hemodynamics and clinical status, milrinone was successfully weaned, and ivabradine was transitioned to metoprolol. In the outpatient setting, GDMT was further titrated. There was slow evidence of reverse remodeling. LVEF remained severely reduced on the echocardiogram 28 months after the initial diagnosis, with normalization at 52 months (Videos S1 and S2). The patient is currently NYHA Class 1, not requiring loop diuretics, and has not had a HF admission in >4 years.

## 4. Patient 3

A 44-year-old male was admitted with months of HF symptoms. Workup revealed moderately dilated LV, EF 19%, no edema or LGE on MRI, normal RV size, mildly reduced RV function, and normal coronaries. The etiology was possibly idiopathic, although alcohol may have been a secondary contributor. He was optimized and discharged on GDMT including metoprolol succinate and sacubitril–valsartan.

Despite outpatient titration of metoprolol to 150 mg daily, he had a persistent resting sinus tachycardia of 100–115 bpm for the following months. No secondary causes were identified. He was readmitted 2 months later with low output HF and AKI in the setting of metapneumovirus. Creatinine was 1.57 mg/dL. Initial hemodynamics were right atrial pressure 15 mmHg, pulmonary artery pressure 56/30/39 mmHg, pulmonary capillary wedge pressure 30 mmHg, and Fick cardiac index 1.3 L/min/m^2^. He required milrinone at 0.25 mcg/kg/min but was successfully weaned after decongestion and optimization, with resolution of AKI. He did not tolerate his prior dose of metoprolol, which worsened his hemodynamics and symptoms. His chance of reverse remodeling was poor given chronic resting sinus tachycardia, especially considering the need to downtitrate his beta-blocker. Ivabradine 5 mg twice daily was added and titrated to 7.5 mg twice daily 1 week later, with improvement in HR to 70–80 bpm for the first time in months.

GDMT was titrated over the following months. He experienced phosphene which was tolerable and did not lead to medication discontinuation. His resting HR improved to 60–70 bpm. Echocardiogram at 7 months showed significant improvement. He was transitioned off ivabradine back to high-dose metoprolol. The patient is currently NYHA Class 1, not requiring loop diuretics, and has not had a HF admission in >5 years.

## 5. Discussion

A minority of patients with new-onset HFrEF have a clinical course that leads to LVAD or heart transplantation during or soon after the index admission. For patients dependent on mechanical circulatory support (MCS), there are limited alternatives. Using LVAD as a bridge to recovery and explantation is an option, as seen in the RESTAGE-HF trial [1]. These patients had a relatively short duration of cardiomyopathy of 20.8 ± 20.6 months, in addition to other favorable factors. A less invasive, dischargeable device in the form of Impella BTR may be a future option, with a feasibility study currently underway. In those who are not MCS-dependent, every means to promote native HF recovery must be utilized while simultaneously not missing the “window of opportunity” for advanced HF therapies in those who continue to worsen.

All three of our patients demonstrated favorable signs for reverse remodeling [2] including younger age, new-onset disease, nonischemic etiology, absence of LGE, wide LBBB (in two patients), and absence of familial dilated cardiomyopathy (DCM). Despite their tenuous status including failing inotrope wean (two patients) and complete intolerance of beta-blockers (two patients) or tolerance to only small doses (one patient), we utilized home inotropic therapy (two patients) and ivabradine (three patients) to maintain stability and lower HR and myocardial oxygen demand while optimizing GDMT (three patients) and device therapy with CRT (two patients). This strategy was successful, and all patients demonstrated significant echocardiographic improvement, achieved NYHA Class 1, and avoided HF admission for years. No major adverse events occurred. One patient experienced phosphenes, a known side effect of ivabradine, that he tolerated. One patient had an episode of AF, a known association with ivabradine, although this may also have been related to inotrope and underlying cardiomyopathy. During the initial outpatient period, patients were monitored closely for signs of needing escalation to LVAD or heart transplantation including worsening functional status, escalating diuretic requirement, recurrent HF admissions, intolerance of GDMT, cardiorenal interactions, and other signs.

Chronic inotropic therapy is usually limited to bridge to advanced therapies or palliation. In patients who have favorable factors that predict recovery, it can be used very selectively in those with low-output HF to improve stroke volume and maintain stability over months until they can reverse remodel with aggressive titration of GDMT. The combination of long-term milrinone with beta-blockers is beneficial [3], but its combination with ivabradine represents an attractive alternative until clinical status is more stable. Although chronic inotrope was associated with increased mortality in some of the historic trials, this may be related to the higher doses of inotrope, concomitant utilization of a second calcitrope in the form of digoxin, higher historic targets for digoxin levels, and trials conducted in the prebeta-blocker and predefibrillator eras [4].

Sinus tachycardia is a poor prognostic sign in HFrEF. In the acute setting, it is compensatory, although this initial beneficial response becomes maladaptive. The SHIFT trial [5] suggests that HR may be a therapeutic target. Ivabradine is a selective sinoatrial nodal channel blocker that has a rate dependent negative chronotropic effect without decreasing myocardial contractility. It carries a Class IIa indication for HFrEF with a resting sinus *rate* > 70 bpm in the ESC and ACC/AHA guidelines. In the era of quadruple therapy, additional HF medications can result in a financial burden to the individual or the healthcare system and contribute to nonadherence and polypharmacy. In such situations, it is important to determine the “super responders” who derive the largest benefit. Data from the original SHIFT trial as well as a subsequent subgroup analysis showed larger benefits with a higher resting HR of >77 bpm [5] or >75 bpm [6]. Our patients had a resting HR of 100–120 bpm which may potentially be a group that derives a larger benefit. The average resting HR of the landmark beta-blocker HFrEF trials was consistently <85 bpm. Patients with new-onset DCM with maladaptive sinus tachycardia likely have far more to gain. This phenomenon was observed in the earliest documented case series of beta-blockers in HFrEF by Waagstein et al. in 1975 [7]. Despite an advanced HF phenotype, these patients were naïve to neurohormonal blockade, had an average resting HR of 98 bpm, and demonstrated significant clinical and structural benefits with beta blockade.

We aimed to wean ivabradine once the clinical status of our patients had improved for the following reasons: (1) they eventually tolerated beta-blockers, reducing the benefit of ivabradine which is driven primarily by HR reduction as opposed to the antiadrenergic effect and (2) the cost of ivabradine can be expensive in some countries. We did not see rebound tachycardia upon withdrawal, and HR remained below the guideline recommendation for the initiation of ivabradine.

Reverse remodeling is not limited to the first few months following diagnosis. This concept was illustrated in an analysis of the PROVE-HF study, which showed that sacubitril–valsartan provided structural benefits even in those >1–5 years after HFrEF diagnosis [8]. Reverse remodeling was seen beyond 6 months in de novo HFrEF in the HF-OPT study [9]. In two of our patients, the cardiomyopathy remained severe for ~2 years, with significant improvement taking ~4 years.

## 6. Conclusion

In summary, as we strive to achieve “heart recovery,” we must continue to evolve our strategies, especially in new-onset HFrEF. We describe the very selective utility of home inotrope and ivabradine as a bridge to optimizing GDMT and eventual myocardial recovery.

## Nomenclature

AF: atrial fibrillation
AKI: acute kidney injury
GDMT: guideline-directed medical therapy
EF: ejection fraction
HF: congestive heart failure
HFrEF: heart failure with reduced ejection fraction
HR: heart rate
LBBB: left bundle branch block
LGE: late gadolinium enhancement
LV: left ventricle

## Supporting Information

Supporting Information 1 Video S1 Cardiac MRI at initial diagnosis (Patient 2).

Supporting Information 2 Video S2 Echocardiogram 52 months after diagnosis (Patient 2).
